# Operating in recurrent crises: a qualitative study of decision-making and unintended consequences in a peripheral hospital

**DOI:** 10.3389/fpubh.2026.1728146

**Published:** 2026-03-13

**Authors:** Tomer Bernstine, Michael Edelstein, Sivan Spitzer

**Affiliations:** Azrieli Faculty of Medicine, Bar-Ilan University, Safed, Israel

**Keywords:** crisis, decision-making, health system resilience, hospital management, organizational learning, unintended consequences

## Abstract

**Background:**

During crises, decision-makers often operate under uncertainty, relying on limited evidence and intuition, increasing the risk of unintended consequences. In Israel, hospitals have recently faced back-to-back crises: the COVID pandemic and the war following October 7th 2023.

**Objective:**

This study aimed to explore hospitals’ decision-making processes during crises and how managing one crisis influenced responses to subsequent crises.

**Methods:**

We conducted a qualitative study guided by a modified version of the World Health Organization’s six building blocks framework, and interviewed 18 key decision-makers in a peripheral hospital. Deductive framework-informed thematic analysis compared decision-making during routine operations versus crises.

**Results:**

We found that decision-making processes narrowed primarily to managing COVID-19, with heightened oversight of COVID wards and increased interactions with external factors such as the Ministry of Health. Although mechanisms to minimize unintended consequences existed during routine times, non-COVID areas were often deprioritized during the crisis, increasing the risk of unintended consequences. Notably, the pandemic served as a catalyst for organizational learning, strengthening staff preparedness and improving operational and logistical capabilities during the subsequent war. Furthermore, interactions established during the pandemic contributed to streamlined wartime decision-making.

**Conclusion:**

Decision makers should capitalize internal and external collaborations developed during crises to sustain them during routine times for better preparedness. Moreover, they should mitigate the risk of unintended consequences in non-crisis-related services, which are particularly vulnerable during emergencies. It could be achieved by regular and systematic real-time evaluation checkpoints in place. Future multi-center studies are needed to assess the transferability of these findings across diverse settings and crisis contexts.

## 1 Introduction

In routine times, decision-makers typically have sufficient time to evaluate alternatives and anticipate both direct and indirect outcomes (1). However, during crises, urgency necessitates rapid decision-making, often guided by intuition, reasoning and prior experience (2, 3). The COVID-19 pandemic illustrated this challenge, as hospital decision-makers were put under immense pressure to manage emergency care while maintaining routine services, often lacking sufficient evidence to guide their actions (4).

Although pandemic preparedness plans (PPP) exist at the hospital (5) and health-system levels (6), their effectiveness in addressing novel outbreaks is constrained by reliance on prior events that differ in nature and scale. A Czech multicenter study, reported that despite existing preparedness plans, in response to the COVID-19 pandemic, 72.3% of hospitals developed new policies in real-time (7). Similarly, in England, although 71% of the hospitals included in the study had PPP, they were insufficient for the complexity of COVID-19. This led to unanticipated shortages of protective measures, testing supplies and staffing (8). Comparable gaps were observed in Jordanian hospitals (9). These examples highlight difficulties in the implementation and utility of PPP, such constraints may have caused trade-offs that hinder effective responses, thereby contributing to unintended consequences.

Unintended consequences in healthcare are the additional effects (positive, neutral, or negative) of interventions targeting a specific issue (10). Since healthcare systems are complex and adaptive, system-wide interventions may produce, nonlinear, unpredictable downstream impacts (11). This is partly due to the interactions and interdependence of multiple components (12). For example, during the COVID pandemic, governments implemented non-pharmaceutical interventions (e.g., lockdowns, social distancing). Though intended to reduce hospitalizations of COVID-19 patients, they also resulted in disrupted care for patients managing chronic conditions. This led to a median reduction of 42.3% in healthcare visits and a median decrease of 28.4% in hospital admissions (13, 14). It remains unclear how hospitals accounted for such unintended consequences, monitored and responded to them in their crisis decision-making processes.

Real-world decision-making processes guiding hospital-level responses during the COVID pandemic remain limited. An Italian multicenter study involving 49 hospital CEOs revealed that leaders focused on specific hospital domains and challenges (emergency room, COVID wards and infectious disease departments). Decision-making processes became more centralized, involved fewer actors, and decreased lateral communication (15). A study in five Danish hospitals highlighted a shift in management configurations, with improved learning processes within hospitals throughout the initial phase of the pandemic. As the pandemic progressed, coping and regional collaboration also increased (16). While both studies address various aspects of decision-making processes and coping mechanisms, they reflect leadership perspectives and do not explore how decisions affected other areas of the hospital. A system-wide view that includes interactions among various stakeholders in the organization is needed.

Peripheral and rural hospitals face additional challenges during crises. A Swedish study found that while rural decision-makers felt generally prepared for disasters, they struggled with geographical isolation and limited regional collaboration (17). In the United States, a national survey showed that 95% of rural hospitals could be overwhelmed by approximately 10 patients requiring emergency care during a disaster, indicating their limited crisis capacity (18). Compared to tertiary urban hospitals, peripheral hospitals often face distinct challenges such as difficulties in recruiting specialized healthcare staff, lower institutional prestige (19, 20), limited resources (21) and long travel times that may reduce healthcare utilization (22). These factors underscore the need to study crisis responses within peripheral hospital settings, as findings from large urban hospitals may not be generalizable, especially when considering crisis management and the emergence of unintended consequences.

Israel offers a unique context for examining health system responses to crises. Like every other country, Israel was deeply affected by the COVID-19 pandemic. In addition, over the past two decades, the country has also faced several large-scale conflicts including Second Lebanon War (2006), Operation Protective Edge (2014) and most recently, the October 7th war (2023–2025). Response of hospitals to the October 7th war has been well-documented since its onset (23), as have policies, management and lessons for Israeli hospitals’ trauma system during wartime (24). Although differing in origin, pandemics impose constraints on healthcare systems similar to those imposed by wars globally: In Sudan, 30% of public hospitals ceased operations within 1 year of the outbreak of war (25). In Yemen, hospitals’ supply chains were disrupted (26), while in Syria, attacks led to a marked decline in outpatient visits and deliveries (27). Ukrainian hospitals also exhibited increased emergency department operations, other routine in-hospital services decreased (28). A similar reduction in inpatient and outpatient services was observed during both pandemics and war, as observed in a tertiary care hospital in Ethiopia (29).

Israel’s health system operates under a national health insurance law and provides free-of-charge care at the point of service. The Ministry of Health (MoH) owns and operates approximately half of the hospitals (30), and serves as a central regulator (31). This study was conducted in a secondary hospital located in Israel’s periphery, a region characterized by socioeconomic disparities, limited hospital capacity, and lower ratios of physicians compared to the central region (30). During the COVID pandemic, the hospital restructured its services and redirected logistical and human resources from other departments to COVID wards. Following the onset of the October 7th war, the hospital once again underwent organizational changes, relocating departments to underground levels in anticipation of potential bombing and a surge of injured soldiers and other trauma patients. Both crises were accompanied by reduced patient arrivals, driven by fear of infection, lockdowns, concerns about travel and evacuation from conflict zones. These repeated crises make Israeli peripheral hospitals a relevant setting for exploring organizational decision-making, unintended consequences, and cross-crisis learning, an area that remains largely unexplored area of research.

Although hospital decision-making during pandemics has been described, how unintended consequences are considered, evaluated and addressed remains poorly understood. Decision-making and unintended consequences have not been investigated beyond leadership perspectives, particularly for peripheral hospitals experiencing consecutive crises. Given the complexity of health systems and the interdependence of various stakeholders, a systems perspective is essential (32, 33). Our study aimed to address these knowledge gaps by utilizing the unique setting of a peripheral hospital in Israel that experienced both crises in short succession. We focused on inter-sectoral interactions, key considerations, and strategies to mitigate unintended consequences. This study may enable policymakers to better anticipate and mitigate unintended consequences in future crises.

## 2 Methods

### 2.1 Study design and conceptual framework

We conducted a qualitative study using semi-structured interviews and thematic analysis informed by an adapted World Health Organization (WHO) six Building Blocks (6BB) framework. The aim of the study was to understand hospital decision-makers’ perceptions across administrative and clinical leadership regarding differences and similarities in the decision-making processes during routine times, the COVID-19 pandemic, and the October 7th war. We examined interactions among internal and external stakeholders, and the considerations guiding their decisions. Finally, we explored how intended and unintended consequences were anticipated and mitigated, and whether organizational learning from one crisis was applied to the next. We chose this approach to provide detailed, multi-perspective insights across contexts.

**Figure 1:**
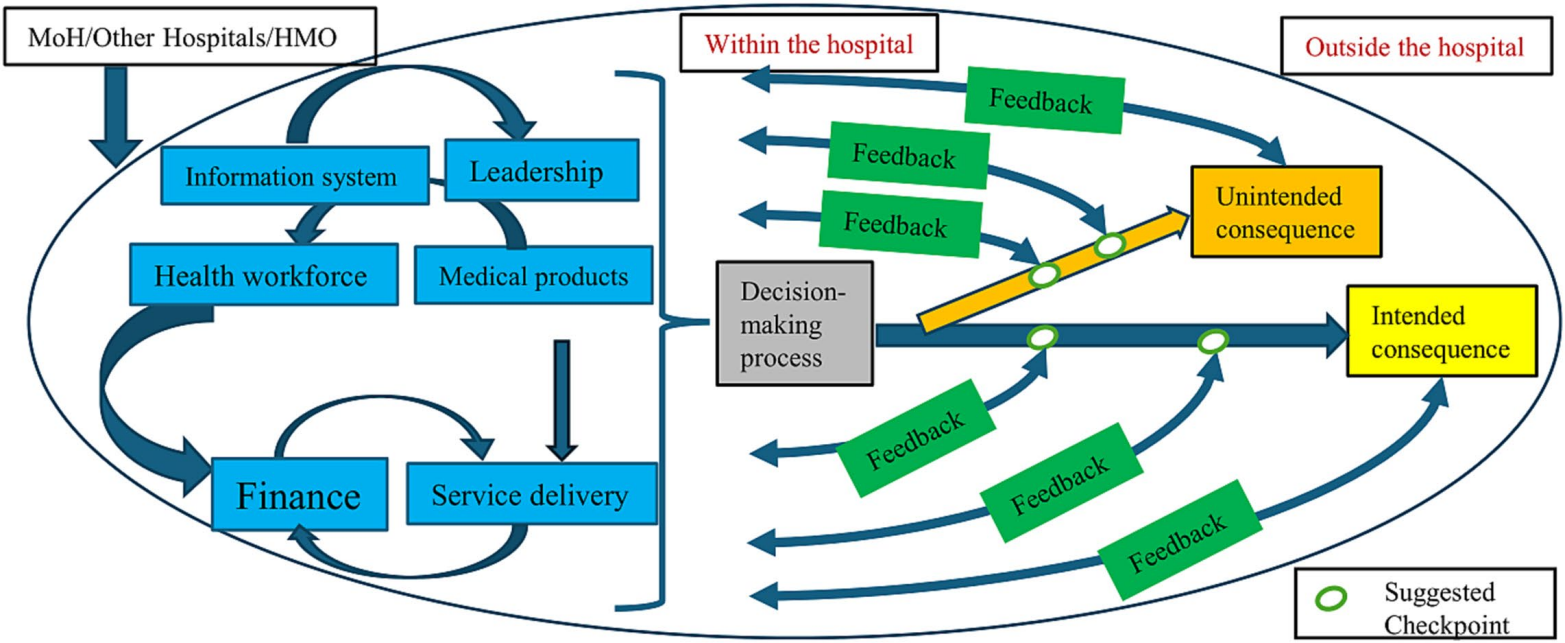
The modified conceptual framework for decision-making processes and its intended and unintended outcomes for hospitals in routine times.

The interview guide was based on the modified WHO 6BB framework. The original framework, published in 2007, conceptualizes health systems through six core components: service delivery, health workforce, health information systems, medical products and technologies, finance, leadership and governance. The interdependence and interactions among these elements were emphasized, while aiming to achieve improved health, efficiency, responsiveness and financial risk protection (32). Subsequent modifications incorporated the influence of external stakeholders (34). Furthermore, the framework was expanded to include both formative and summative evaluations, incorporating feedback for interventions, while applying systems thinking. Hence these adaptations linked operational performance with leadership decision-making which accounted for both intended and unintended consequences of organizational decisions (33). These developments addressed routine operations at the national health system level. We illustrated these adaptations in a graphical manner (Figure 1).

Building on the national-level adaptations, we analytically modified this framework to address a gap in emergency management at hospitals. Although structured WHO framework assists emergency responses (35), there are limited frameworks for identifying and mitigating unintended consequences in hospitals. We therefore adapted the modified framework to capture and mitigate unintended consequences during crises at the hospital level. In our model, each of the WHO 6BB is represented intra-organizationally within the hospital. We examined interactions among these components and how they impact hospital decision-making processes. As interventions were implemented following these decisions, we sought to incorporate the existence of formative and summative evaluations of intended and unintended outcomes. We also assessed inter-organizational interactions such as those with the MoH or other hospitals, and their influence on internal processes. Additionally we introduced the concept of “checkpoints,” defined as structured time points designed to evaluate and assess both intended and specifically unintended consequences (Supplementary Table 1) (32–34, 36).

### 2.2 Participant recruitment and data collection

Semi-structured interviews were conducted from March to September 2024 with decision-makers and administrators for each of the WHO 6BB at the hospital and department levels (COVID and non-COVID, Table 1). Interviewees were purposively identified and recruited by the research team through professional acquaintances and relationships with hospital administrators. This process was supplemented by publicly available hospital records. Subsequently, we invited relevant stakeholders to participate in the study. The interview guide was first piloted with one administrator and then refined. Questions focused on decision-making processes with prompts to guide the participants toward our research objective. The second and third authors utilized their experience in health services research and qualitative methodologies to refine the questions. Interviews were conducted with assurances to participants that anonymity would be maintained to promote genuine responses and perspectives. Given the relatively small number of peripheral hospitals in Israel, efforts were made to present responses in a generalized manner to prevent identification of participants. Participants were presented vaguely and according to the 6BB to preserve anonymity.

**Table 1:** Study participants.

Building block representation	Number of interviews
Leadership/Governance	4
Finance	1
Information system	3
Health workforce	2
Medical products, vaccines and technology	1
Service delivery	
COVID	2
Non-COVID	4
Monitoring and control	1

Interviews began with the participants describing the decision-making processes in routine times to enable a comparison of these processes during crises. We then asked participants to explain the process of opening a new clinical service in the hospital, which was used as a proxy example for a practical example to the planning and construction of the COVID department. Participants were then asked to describe the same processes during the COVID pandemic and the October 7th war (Supplementary Table 2). Each interview lasted between 20 and 60 min. Interviews were conducted in Hebrew and held face-to-face, apart from three participants whose interviews were conducted via Zoom.

### 2.3 Data analysis

Each interview was recorded and manually transcribed. Data were analyzed using a deductive thematic analysis, guided by the modified 6BB, and a codebook was derived accordingly. The analysis followed the phases of thematic analysis: familiarization with the data, generating initial codes, searching for themes, reviewing and defining themes (37). Analysis began with the research team (first and third authors) reading and re-reading transcripts to familiarize themselves with the data. To enhance analytic rigor, a subset of interviews (*n* = 3) was double-coded by two of the authors to achieve inter-rater reliability (Supplementary Table 1). Transcripts were uploaded into ATLAS.ti (version 24.0.0.2957). Quotations used to exemplify themes were translated using generative AI (ChatGPT-5.2 (38)) from Hebrew to English. The thematic structure was predefined utilizing the modified 6BB framework. Interview data were deductively coded and mapped to the pre-defined themes to examine decision-making processes and health system operations in routine and crisis periods. We chose this analytic method to enable systematic examination of the data, in relation to the modified 6BB, thus we ensured comparability across different periods. Translations were verified and edited where needed by the authors. In this study, the sample was naturally bounded: all eligible participants within the organization were interviewed (with the exception of one participant). Because the entire population was included, the goal was not theoretical saturation through ongoing sampling, but rather comprehensive coverage. While traditional data saturation refers to sampling until no new codes or themes emerge, this approach is less applicable within a bounded organizational setting. Given that our aim was to understand a complex phenomenon involving multiple stakeholder perspectives, meaning saturation was prioritized over coding saturation. As suggested by Hennink et al. (39) we were able to achieve meaning saturation through full inclusion of all relevant participants. Consistent with the concept of information power described by Malterud et al., full inclusion within a well-defined population ensures maximal variation and minimizes the risk of missing perspectives (40).

### 2.4 Researchers positionality

The interviewer (first author) was trained in qualitative research and had no professional relationships with the interviewees, except for one individual, with whom there was a prior professional acquaintance. Second author had extensive professional relationships with many of the participants, which enabled access to those decision-makers. To avoid bias, the second author was not involved in the collection or management of the interview data. The third author had professional relationships with two of the participants, which facilitated their agreement to participate. During data analysis, the second and third authors were blinded to interviewees’ identities. Despite these measures, we acknowledge that researchers’ backgrounds and partial relationships may have influenced interpretation. This potential influence was addressed through reflexive discussion.

### 2.5 Methodological rigor

Methodological rigor was ensured through several strategies. Prior to interviewing, the first author engaged with participants to build trust and increase openness. This approach was chosen to lessen concerns about being judged for their actions during crisis. Analysis and coding were conducted using a systematic deductive approach to limit subjectivity and increase reproducibility. Transferability was addressed by providing rich descriptions of the organizational context, decision-making processes, and crisis settings, enabling readers to assess the applicability of findings to other healthcare systems or emergency contexts. Dependability was supported through the use of a predefined, framework-based interview guide adapted from the WHO 6BB framework. Confirmability was strengthened by consulting two independent peers with experience in qualitative research to validate our interpretations.

## 3 Results

Nineteen administrators, nurses and doctors representing the health system 6BB were invited, of which 18 agreed to participate (Table 1). Study participants included both males and females, from diverse ethnic backgrounds, encompassing a range of professional roles and areas of expertise, with varying years of experience in hospitals operations. Some participants held significant roles only during the pandemic, others only during the war, but most interviewees had leadership experience in both crises. This overlap provided valuable insights into the similarities and differences in the decision-making processes across the different crises.

According to the modified 6BB framework, we identified four major themes: (a) the decision-making processes during a crisis compared to routine times; (b) the intended and unintended consequences of decision-making during COVID-19; (c) the learning processes and transfer of lessons from one crisis to another; and (d) crisis management in a secondary-peripheral hospital.

### 3.1 Decision-making processes during crisis compared to routine times

This theme captures the procedural trajectory, including shifts in decision-making logics during crisis versus routine contexts, the range of actors engaged in the processes, and the evolving dynamics of interaction with both internal (representing the 6BB within the hospital) and external stakeholders (representing influences originating outside the health system, as presented in the modified framework). In the hospital, the core decision-making body is comprised of senior leadership, including the Chief Executive Officer (CEO), Deputy CEO, Chief Nursing officer, Chief Financial Officer, Administrative Director, Director of the hospital’s economic development arm, and Head of Human Resources. This core group is routinely supported by input from domain-specific stakeholders, whose perspectives are consulted based on the nature of the issue at hand. Each decision is anchored by a designated point-of-contact, responsible for coordination and follow-through. Under routine conditions, decision-making processes are characterized by structured deliberation and consideration of long-term strategic factors, often balancing service delivery needs with financial viability, workforce capacity, and regulatory requirements. In contrast, crisis contexts such as the COVID-19 pandemic precipitated a marked shift in both the urgency and content of decision-making. Prioritization of time-sensitive responses led to a reconfiguration of typical governance flows, with accelerated input channels, compressed timelines, and greater reliance on operational agility. The dynamic interplay between service delivery, leadership (governance), financial considerations and workforce mobilization, core elements of the WHO’s health system 6BB, was particularly salient during these periods of disruption.

Interviewees emphasized that, in contrast to routine periods characterized by weekly leadership meetings, the decision-making processes during the crisis intensified significantly. Leadership convened multiple times daily to navigate rapidly evolving regulations and the uncertainty of the unfolding situation.

Health workforce #1: *“The decision-making processes involved management meetings on a daily basis, even twice a day. because there were new guidelines on a daily and sometimes hourly basis… new work agreements that enabled us to deploy manpower differently we would make decisions and then communicated those instructions to the staff working in the hospital.”*

This heightened sense of urgency created opportunities to advance initiatives that had previously encountered institutional or procedural barriers.

Leadership/ Governance #1: *“when you are in a crisis it’s easier to get people on board… And I managed during that period to launch a hundred projects simultaneously because yes, people were more committed.”*

Under routine conditions, decisions regarding the initiation of new clinical services were informed by considerations such as the needs of at-risk populations, the expertise of clinical staff, existing infrastructure, financial feasibility and regulatory approval. In contrast, during the crisis, decisions were made more rapidly, and financial considerations were notably deprioritized due to the urgent demands of the situation. Healthcare workforce was added to mitigate the crisis with rapid approvals by the regulator.

Leadership/ Governance #1: *“In a crisis, economic considerations are almost non-existent. I mean, they exist but they are less disruptive. Then the ministry (of Health) always says we will support you.”*

Medical products, vaccines and technology #1: *“In routine the process is long… and there’s really a process of recruiting manpower…until you get the recognition (by the MoH) (During wartime) there is a kind of a green lane for setting up a unit…it means that there is an easier process by the ministry that allows for progress …infrastructure is done on the go, manpower recruitment and training are conducted on the go.”*

Hospital staff indicated that the COVID-19 pandemic drastically reshaped internal stakeholder interactions, intensifying communication and collaboration due to the demand creating strong interprofessional ties for rapid decision-making.

Health workforce #1: *“Adjustments always need to be made… This is why the communication and the synchronization between* var*ious stakeholders is very, very important. especially when we need to respond quickly and with urgent changes.”*

While internal coordination among hospital stakeholders intensified during the crisis, there was also significant enhancement in cross-organizational collaboration with external actors, notably the MoH and neighboring hospitals. This outward-facing synchronization reflected an adaptive reconfiguration of governance, service delivery, and information systems, key components of the WHO’s health system 6BB, allowing for more cohesive system-level responses. However, these inter-institutional linkages tended to diminish once the immediate crisis subsided, as organizations reverted to more routine operational modes. Importantly, the collaborative infrastructures and relational capital established during the COVID-19 pandemic served as a foundation for more efficient coordination with regulatory bodies in subsequent emergencies, such as the October 7th war.

Leadership/ Governance #1*: “We were constantly in touch, asking ‘What are you doing?’ We constantly talked, the 25 hospitals spoke a lot with each other. Which, by the way, has been greatly missed since the end of COVID…. It returned a bit after the seventh of October (October 7th war) for about 2 months… and it ended again.”*

### 3.2 Intended and unintended consequences of decision-making during COVID

#### 3.2.1 Intended consequences

During the pandemic, the hospital underwent a substantial operational shift, redirecting its focus from routine service delivery to the urgent demands of managing the COVID-19 crisis. *‘Suddenly you are in a place where you need to react. It’s the stable, beautiful ship that suddenly you need to turn quickly so that it does not hit an iceberg.’* (Leadership/ Governance #3). This abrupt transition necessitated the rapid development of feedback mechanisms and continuous formative evaluation, particularly within the newly established COVID-19 units. Initially, there was heightened organizational learning concentrated on the emergent needs of these departments, supported by regular input loops and performance monitoring. However, as the pandemic progressed the hospital adjusted and balanced emergency as well as routine healthcare services. The initial acute phase was marked by a strong sense of urgency and a reduction in healthcare demand. However, during the prolonged phases of the crisis, hospital leadership and frontline staff adapted so that they can continue providing care for patients with non-COVID related conditions. Consequently, modifications and the gradual reopening of routine services were implemented to accommodate those needs. Over time, as the organization acclimated to this new operational landscape, the frequency and intensity of evaluative processes to COVID wards also diminished.

Leadership/Governance #2: *“During the first wave and lockdown, there wasn’t a problem, because the public wasn’t supposed to leave their homes, the need to come to the hospital decreased… there was decreased demand alongside decreased capabilities, because we wanted to move the doctors and nurses to the COVID (wards)… But as the emergency prolonged - COVID for 3 years, war for 11 months… you cannot close routine service and tell patients “I do not have (routine services), I’m waiting for a war” Therefore, you always need to make these balances.”*

Within the COVID-19 departments, there were organizational learning processes which enabled implementation of changes throughout the operational phase (representing the evaluations of interventions and reflecting the application of systems thinking within the modified 6BB). These mechanisms aimed to enhance both the quality of patient care and the wellbeing of the healthcare staff. Periodic satisfaction surveys were administered to capture staff experiences and identify areas for improvement, creating formative and dynamic evaluation of organizational learning under crisis conditions.

Service delivery #2: *“…(regarding COVID department), we would talk and discuss with the head nurse and the department manager. And each time a [COVID] department closed, there was a requirement from management to report what to preserve and improve. Not just from COVID ward, but from all departments. So the second time we opened (COVID ward), according to what the staff said, we handled it better.”*

The unique position of the interviewee “Service delivery #2” within the hospital may have provided him with a broader organizational perspective of the entire hospital, which could potentially explain his reference to evaluations of non-COVID departments, an aspect not mentioned by other interviewees.

#### 3.2.2 Unintended consequences

Under routine conditions, decision-making processes are typically deliberative, supported by institutional mechanisms that allow for slower implementation and structured assessment of potential consequences. These mechanisms include systematic feedback loops, stakeholder engagement, and the incorporation of diverse professional perspectives. In contrast, during crisis situations, decision-making shifts toward rapid response, prioritizing immediate operational needs. As a result, less attention was initially given to the unintended consequences of these decisions.

Health workforce #1: *“…(In routine), we build scenarios and try to minimize the unexpected as much as possible…and thinking as broadly as possible…. everyone who is supposed to be affected by the decision is included in the discussion… To really understand the implications of the decision on the* var*ious angles… And yet, there are things that are unexpected and they happen, and then they are dealt with again. (in crisis), our focus is much more specific, we are primarily focused on the emergency…obviously there are also effects on other areas but the immediate focus is on dealing with the emergency.”*

Despite the urgency of crisis response, several unintended consequences emerged, although no formal evaluative mechanism was in place to systematically examine them. One such example is the ambivalent perceptions regarding the status of non-COVID healthcare staff. Some administrators expressed concern that these professionals were marginalized or overlooked in institutional priorities, contributing to feelings of neglect. Conversely, others believed that non-COVID departments were relatively advantaged, noting that their workload was reduced while staffing levels remained largely unchanged. From the hospital administration’s perspective, the diminished demand for non-COVID services during the pandemic reduced the operational risks in these areas, thereby potentially limiting adverse consequences.

Medical products, vaccines and technology #1: *“… I remember that they actually felt neglected… That the attention was only directed toward the COVID departments, and they kind of you know, COVID departments being like an elite unit [in the army], and the others being the rank and file.”*

The hospitals’ approach to risk management regarding unintended consequences was evident during its response to the pandemic. Leadership demonstrated awareness of potential harms to patients’ wellbeing during hospitalization, such as reduced services and limited staff availability, resulting from emergency reorganizations. Yet, they emphasized maintaining quality of treatment and ensuring that critical services continued despite these constraints.

Service delivery #2: *“You cannot prevent all risks… you know you might harm other departments. You know you are affecting the quality of service overall, and you are still willing to accept the impact on service experience for the sake of treatment quality…”.*

To effectively evaluate and assess unintended consequences, structured feedback by the clinical non-COVID teams is essential. Such feedback may arise bottom-up, as teams “raise flags,” or top-down, through proactive management assessments and interactions. In our setting, despite the absence of formal top-down or bottom-up clinical assessment of possible unintended consequences, non-COVID teams were still able to voice their concerns to the administration. Although administrative leadership did visit these departments, these visits were less frequent compared to those in the COVID department. This prioritization toward immediate crisis response potentially reduced the visibility of other clinical services. The lack of a formal feedback mechanism does not preclude the possibility of clinical and professional unintended consequences arising within these departments.

Finance #1: “*(regarding feedback of non-COVID departments). It does not reach the management every day (laughs), of course not… this is something unique (COVID pandemic and wards), that requires the full attention of management on a daily basis.*”

### 3.3 Learning processes and transfer from one crisis to another

The experience of managing the pandemic had a positive effect on the hospital’s management decision making practices, strengthening capacities, and enabling effective response during war-time. Common characteristics of both the COVID pandemic and war led to easier adaptations in service delivery and logistical operations. The transition from one crisis to another catalyzed organizational learning, especially in the realm of staff preparedness. Interestingly, while some administrators saw similarities between the two crises, drawing conclusions from one crisis to another, others did not.

Leadership/ Governance #2: *“During COVID we developed a good understanding of the hospital’s infrastructure, in terms of resource capabilities. In terms of the reserve capacity (of staff to work during crises), in terms of staff training.”*

Medical products, vaccines and technology #1: *“(about similarities or differences between COVID and war) Definitely similar, an emergency is an emergency. It could be COVID, it could be war, it could be an earthquake.”*

Health workforce #2: *“(about similarities or differences between COVID and war) Completely different, completely different. Look, if there was an intense war right now, then maybe I would say it is like COVID, I’d split my teams into two shifts, one working 12 h and the other 12 (hours). But in the current situation, I do not see any comparison at all between them.”*

Effectiveness of managing crises depends on granular operational adaptations that support frontline staff. During the COVID-19 pandemic, challenging working conditions occurred which caused difficulties for frontline staff. To sustain healthcare workers’ ability to provide care during the following crisis, systematic support mechanisms from logistical to psychological resilience services were developed. These measures emerged due to organizational learning by the leadership to address staff and their families’ wellbeing. The aspect of team resilience was amplified post-COVID, as leadership invested in the matter.

Medical products, vaccines and technology #1: “*How to prepare for 12-h shifts instead of a single 7-h shift. How do you prepare to take care of (staff) families, patients, and employees’ children to enable staff to come in and work here. There are many lessons at this micro-tactical level….”*

Additionally, the interactions established with the MoH during the COVID-19 pandemic contributed to a more streamlined decision-making processes in the subsequent crisis.

Finance #1:*”…I want to have financial security to avoid putting the brakes on something that needs to happen rapidly. We really learned this after COVID… during the war we already knew how to manage, knew whom to contact, knew where to get the budgets from.”*

### 3.4 Crisis management in a secondary-peripheral hospital

The hospital’s peripheral and secondary-level status was repeatedly referenced by interviewees as a defining contextual factor. Compared to tertiary-care hospitals in the center of the country, the hospital routinely operates with limited resources. The MoH provides financial support and allocates budgets to hospitals in accordance with the National Insurance Law and activity-based procedures. As the study hospital serves a smaller patient population compared to tertiary urban centers, its routine budget is correspondingly lower. However, during crisis conditions, regulatory authorities placed targeted emphasis on bridging longstanding gaps. The heightened visibility and urgency associated with the emergency context prompted accelerated support from the MoH, enabling resource allocation and system adjustments that would have been less feasible during routine periods.

Finance #1: *“One of the major achievements of the pandemic was that it helped reduce the gaps. These gaps were very very large… but they narrowed. in infrastructure, facilities, and human resources… And now, with the war, it’s the same.”*

The hospital’s unique setting served both as a limiting and a facilitating factor; its geographic location was considered a drawback, given the long distance from other hospitals in the region and closeness to potential battle fronts. As a result, the hospital conducted drill-scenario as an isolated, standalone facility and how to manage care in that context. Its relatively small size enabled personal familiarity among different stakeholders, which contributed to managing the crisis more efficiently. Moreover, during COVID pandemic the spread of the virus which primarily began in the center of Israel, provided temporal advantage to prepare for the peak of the virus in the periphery. Yet the hospital’s size adversely affected ambulatory services due to insufficient staff handling of its tasks, as resources were shifted toward inpatient and emergency care.

Medical products, vaccines and technology #1: *“In this context, there is an advantage to the small number (of staff), that you know everyone and this really allows you to maneuver and achieve an absolute level of control, gap management, and task monitoring, which is simpler.”*

## 4 Discussion

Our study describes insights into intra- and inter- hospital decision-making processes in an Israeli peripheral hospital during two successive crises: the COVID-19 pandemic and the October 7th war. While prior research has described hospital responses to a single crisis (16, 41) our study captures the experience of decision-makers who actively managed two distinct crises and how lessons learned from the first crisis were applied to the second.

Our findings indicate that during a crisis, the decision-making processes intensify and include changes in prioritization. The focus shifted from an emphasis on financial considerations during routine times, toward creating the service delivery needed during the crisis (e.g., COVID wards during the pandemic and surgery/trauma during war). Our findings are similar to those of Romiti et al. (15) and Duvald et al. (16), who emphasized how hospitals had to respond rapidly, honing their intra-organizational focus on one aspect (COVID patients), cutting all financial restrictions to achieve care during the crisis (16). Not only did organizational priorities change during the crises, but we also saw an increase in intra-organizational inter-professional communication. This was facilitated through frequent decision-makers meeting and strengthened collaborations with healthcare staff, especially among front-line staff. These collaborations contributed to improved and more streamlined management of both the COVID pandemic and the war. A multi-national study observed that, during the pandemic, top-down communication was less effective in non-COVID compared to COVID departments. Bottom-up communication was limited overall, and this gap was prominent in non-COVID compared to COVID departments (42), our results align with these findings. By distinguishing between COVID and non-COVID departments, we identified variations in top-down collaboration depending on the crisis focus. A key strength of our findings is that these communication and coordination patterns were observed during both emergencies, and not an isolated event.

In addition to internal intensified coordination and communication, we also found increased interaction with external actors (e.g., MoH and neighboring hospitals). In Israel there is no single governmental law that guides hospitals on how to operate under prolonged emergencies, but there is a standardized system coordinated by the MoH (43). In addition, hospitals are instructed to prepare toward “all-hazard” emergency scenarios (Mass casualties events, biological events) with similar principles for those crises and modify only components that are hazard-specific (allocating life-saving equipment, preparing infrastructure, assigning staff to relevant sites) (44). In order to prepare for disasters, hospitals are required to participate in mandatory exercises and drills. Their preparedness plans are based on national guidelines as well as the recommendations of each hospital’s multi-hazard disaster preparedness committee (23, 43–45). Furthermore, a recently published study regarding the Israeli health system showed relatively similar patterns of organizational resilience in hospitals after the October 7th war, perhaps due to constant preparedness for emergencies, which swiftly transitions from routine to a crisis mode. This ongoing readiness and adaptability likely contribute to their high level of organizational resilience (46). Some of the lessons learnt during COVID pandemic were applied in some Israeli hospitals during the October 7th war, in particular the management of mass casualties, which included engaging with various stakeholders from logistics, clinical and human resources aspects to conduct frequent assessments on how to best manage the injured arrivals (45). Amplified collaboration between different hospitals has been noted in other settings as well (16, 41, 47). Sydnes et al. (48) review highlighted that interorganizational coordination during emergencies, is influenced by formal and informal organizational communication channels, as well as by organizational structures, norms and leadership. In our analysis, we identified the use of informal communication channels (e.g., WhatsApp groups), alongside more structured interactions, such as monthly inter-hospital meetings. Leadership coordination was not solely under the responsibility of the hospital CEO, instead, multiple stakeholders engaged in parallel consultations with peers at other hospitals. These multi-level interactions helped facilitate a more effective and agile response and management during both crises.

Crises often shift organizational focus and intensify collaboration, to respond for the upcoming emergency. Due to the limited resources available in hospitals, this reorientation frequently results in changes to routine procedures (5), which in turn can trigger unintended consequences. We found that during routine times decisions face fewer time constraints. This allows addressing multiple stakeholders’ perspectives, receiving feedback, and adjusting accordingly to the unintended consequences that emerge. Our results which align with those of Oliver et al. (10), emphasize that addressing unintended consequences requires raising awareness, engaging appropriate stakeholders, and designing and evaluating interventions (10). Monitoring clinical outcomes in non-crisis wards is essential for detecting unintended consequences. However, in our setting, such monitoring was not conducted systematically, unlike in the COVID department, which was subject to continuous monitoring. A study investigating the impact of COVID-19 on routine services in an Israeli hospital, found no significant negative effects on acute neurological or cardiological services (49). This finding points to the potential resilience and complex adaptive responses, which warrant further research.

Another aspect of crisis management that we found is that decision-makers who had experienced one crisis were able to quickly re-establish inter- and intra-organizational actions and abilities during subsequent emergencies. Effective inter-crisis learning requires decision-makers to recognize similarities and differences between these situations and adapt their response accordingly. Hospital resilience depends on the capacity and ability to absorb, adapt, transform and learn. Within an all-hazard approach it is essential to generalize common crisis features (around 80%) while tailoring a limited portion of preparedness and response to the specific characteristics of each upcoming hazard (50). In our setting, both crises shared features such as the need for rapid decision making and intensified intra- and inter-organizational collaborations. Both crises were characterized by major uncertainty regarding the magnitude of the impact. In the initial phase of the pandemic, there was a heightened sense of fear from the unknown and possible high mortality. Similarly, at the onset of the war national forecast warned of possible mass invasion from enemy armies and mass casualty events. Both crises required significant reorganizations, during the war, departments were physically relocated to bomb-proof protected areas, while during the pandemic, COVID departments were created also requiring physical relocation of other departments. Both crises needed prolonged crisis management: the pandemic lasted 2.5 years and the October 7th war persisted for over a year in the hospital’s area. Though intensities varied over time in both crises, with management alternating between routine and crisis modes. Notwithstanding these similarities, important differences existed between the two crises. In Israel, there is longstanding familiarity, preparedness and participation in armed conflicts, whereas the pandemic represented an unfamiliar challenge with high uncertainty. The October 7th war erupted suddenly and unexpectedly, while the gradual increase in COVID incidence over days and weeks provided more time for planning despite significant uncertainty and ambiguity. The pandemic also influenced pre-existing health inequity with minority groups such as the Arab or Ultra-Orthodox populations experiencing higher excess mortality at different times of the pandemic, compared to the general population (51, 52). This is particularly relevant for planning the post-crisis burden of disease in the periphery where the majority of the Arab population resides.

The continuity of leadership, as noted in our study setting, enabled lessons from the first crisis to be applied to the second. This unique factor is not always possible due to organizational staff turnover but highlights the importance of staff retention and institutional memory in organizational planning. Mohtady et al. (53) conducted an integrative review on organizational learning in hospitals following disasters and their impact on resilience. Among the 22 included studies only four completed a full cycle of planning to doing, then studying, and translating into actions. However, none of the hospitals in these four studies examined the impact of those learning processes in real-world subsequent disasters. The opportunity to apply lessons from a previous recent crisis to manage new ones is both rare and challenging. A systematic review by Doran et al. found that, prior to the COVID pandemic only 11 studies had recommendations based on 1918 Spanish Influenza and the 2009 Swine Flu pandemic, mainly focusing on mortality rather than hospital preparedness (54). Rapid changes in medical knowledge, pharmaceutical interventions and healthcare infrastructure limit the value and applicability of lessons from earlier pandemics. Even the 2009 Swine Flu pandemic, which emerged only 15 years ago, was far less severe in magnitude and impact than the COVID pandemic. As climate change and global political instability worsen, the likelihood of scenarios including multiple consecutive or concurrent crises is expected to increase in the near future (55). It is therefore crucial to build mechanisms that enable continuous learning and improvement from one crisis to the next for a better resilient health systems. Our study provides a rare, documented narrative of how the lessons from one crisis were immediately applied to another.

### 4.1 A guiding framework for learning during and after crises

**Figure 2:**
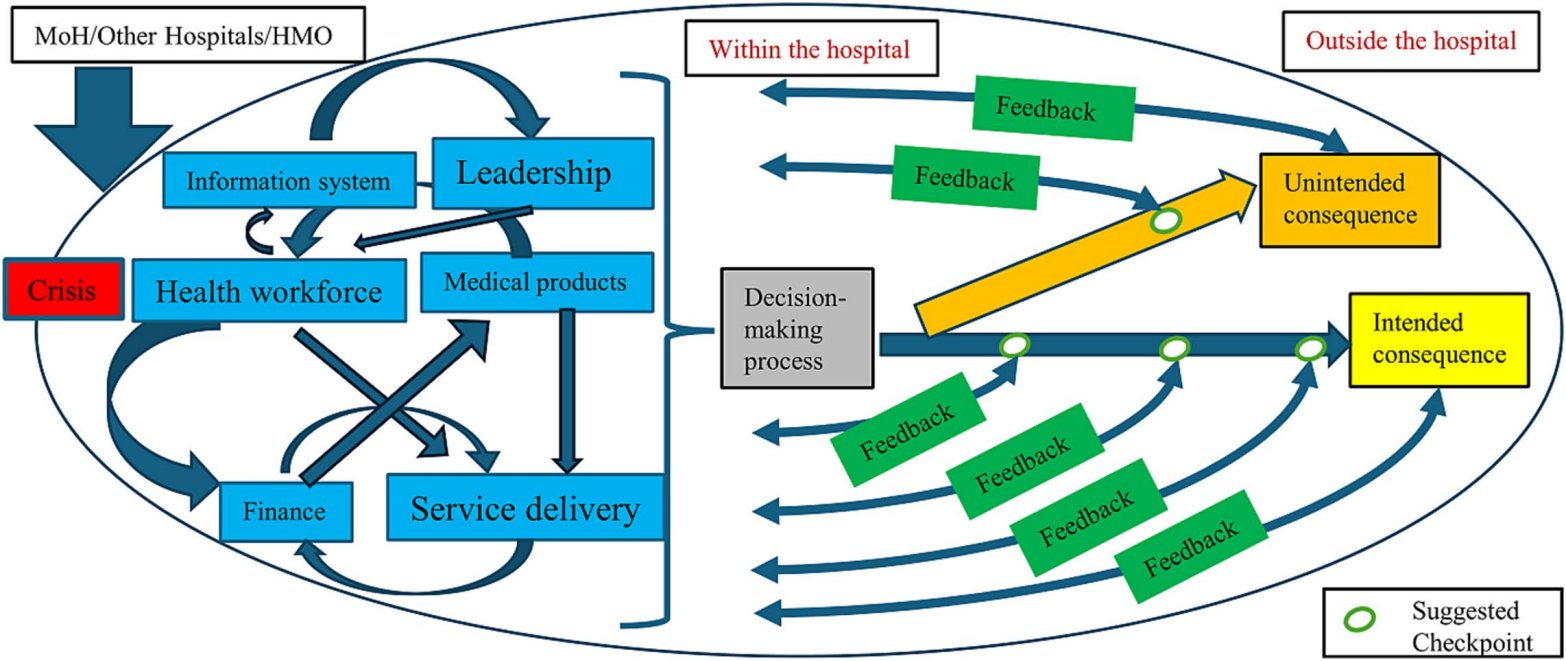
The modified conceptual framework for decision-making processes and its intended and unintended outcomes for hospitals during emergencies.

In our research, we identified key differences between routine operations (presented in the Methods section, Figure 1) and crises in a secondary peripheral hospital, illustrated graphically in Figure 2, these include:

- We observed shifts in the impact of specific constructs during routine times and crisis periods. It was most notable for the Finance building block, as it had a pivotal role during routine times, in contrast, during crisis its influence was relatively lower. It was accompanied by the rise of service delivery and health workforce which were more notable. In the illustrations of the frameworks building blocks are squarely represented with variations in size depending on their influence.
- During routine times, internal organizational interactions as well as external organizational collaborations existed. However, it was found that during crises those interactions intensified both intra- and inter-organizationally. The arrows between the building blocks illustrate intra-organizational collaborations, while those outside the hospital represent inter-organizational collaborations. Both the number and size of arrows increase during crises, reflecting their intensified nature.
- While during routine times feedback and evaluations were conducted at the hospital level for various services, during crises it changed. Those processes mainly addressed specific crisis-related services and occurred much more rapidly and frequently for them. This concentrated focus, was accompanied by reduced feedback in other areas of the system. Feedback loops could be bottom-up or top-down and therefore are represented with double arrowheads.
- During routine times unintended consequences are more endured and mitigated by decision makers. It can be achieved by multiple perspectives of relevant stakeholders. Compared to crises, where those consequences can be more significant since the focus of administrators is less directed toward non-epicenter services. In the illustrations they are presented with larger longer-lasting arrows during crises compared to routine times.

Our modified framework can serve as a practical tool for decision-makers, particularly during emergencies, as the focus logically and desirably shifts to address the most immediate threat. We propose to incorporate real-time feedback mechanisms and evaluation checkpoints for the immediate threat (intended consequences) and non-epicenter “events” (unintended consequences). Since emergency situations can emerge unexpectedly and last an unknown duration of time, a predefined time frame for evaluations should be made. The first checkpoint can be for example, 1 week after the beginning of the crisis, with the next one after 2-weeks, 1- month and so on- exact times should be decided according to the nature of the crisis. Real-time evaluation around those checkpoints should be conducted to evaluate clinical and non-clinical outcomes in all departments and implement corrective measures where needed. Outcome indicators will be crisis dependent but should focus on outcomes that are likely to change in the short run. Examples can include mortality, medical errors, length of stay, referral rates etc. but should be guided by the nature of the crisis. Methodologies to monitor such consequences can vary but can include before-and after studies and descriptive time-series analyses, detecting sudden changes. In addition to the quantitative assessments, perspectives from staff in different roles in each department should be captured, to provide diverse early insights. Tools for rapid qualitative appraisal have been developed and can be used in such context (56). This mixed-methods feedback model combining both top-down and bottom-up feedback can strengthen responsiveness and staff engagement during prolonged crisis.

While unintended consequences may be partially unavoidable during emergencies, implementing checkpoints will enable decision-makers to re-evaluate decisions and assess both potential benefits and harms resulting from crisis-related actions. Checkpoints allow decision makers to adjust actions both formatively and summatively to optimize health outcomes in times of uncertainty. Moreover, this structured mechanism can enable ongoing learning processes during the crisis and not only after it ends.

### 4.2 Policy implications for health system leaders and hospital administration toward future crises

Our findings suggest that emergencies foster a collective focus on shared goals, together with reduced administrative barriers to change, enabling the advancement of novel initiatives. At the management level, maintaining inter-institutional coordination between medical centers and with other stakeholders within the healthcare system during routine times may benefit the quality of routine services and improve preparedness and resilience during crises. At the clinical level, enhanced routine collaboration and communication across clinical departments within different hospitals could improve shared learning and identification of unintended consequences through comparative analyses. Developing and implementing systematic approaches to monitor adverse consequences, both qualitatively and quantitatively, is warranted. Specifically, the following recommendations would improve readiness for crises and reduce the negative impact of unintended consequences when crises occur:

1. Operating tools such as dashboards, on all aspects of hospital functioning, and not only on crisis aspects, when an emergency is declared, will enable real-time monitoring of adverse consequences. Non-crisis wards clinical data will enable to track emerging unintended consequences, guide managerial decision-making, and prevent unnoticed deterioration. Since during emergencies, there is a sense of “call for duty” regarding the upcoming crisis, relying solely on healthcare workers to “raise flags” in non-epicenter wards maybe insufficient.

2 Developing simulation workshops and learning seminars to strengthen feedback loops and decision-making processes.
3 Establishing multi-institutional “crisis memory banks” may promote cross-context learning and long-term system resilience.
4 At the clinical level, enhanced routine collaboration across clinical departments within different hospitals could improve shared learning and identification of unintended consequences through comparative analyses during crises.

### 4.3 Limitations

There are several limitations to our study. First, it was conducted in a single hospital within a specific context, which limits the generalizability of our findings. As the peripheral context was a central aspect of crisis operations, conclusions should be drawn cautiously, especially when considering their applicability to larger urban or tertiary care centers. Second, explaining and eliciting the concept of “unintended consequences” proved challenging, particularly when asking about new clinical services (used as a comparison to opening COVID wards) under routine conditions. Since data were collected in 2024 about scenarios occurring before 2020, responses were susceptible to recall bias. Some participants had not experienced, or did not remember, opening a new service comparable in scope to COVID wards, during routine times. Thus, engaging a hypothetical scenario such as establishing a new hospital clinical unit, may have seemed abstract. In contrast, during crises, questions about non-COVID departments elicited clearer reflections on unintended consequences, likely because events were recent. Differences in response may also reflect the tangible nature of constructing COVID wards, which enabled participants to draw on real-world experiences rather than speculate. Although unintended consequences can be positive, negative, neutral and are inherently unavoidable, interviewees may have tended to minimize or omit negative outcomes to avoid being judged for their actions. Future research should address this bias by refining questions that encourage responses that provide the “entire picture” to ensure comprehensive insights. Finally, the timing of data collection is a limitation. Participants were interviewed during the war, which may have created a recency bias favoring that emergency. We sought to mitigate this by asking the same questions for both COVID and war periods, thereby enabling clearer distinctions between the two events.

## 5 Conclusion

As the likelihood of concurrent or consecutive crises increases in many parts of the world, learning from past crises to better manage future ones is becoming increasingly essential. In this study we examined decision-making processes that occurred in a peripheral secondary care facility in Israel during two consecutive crises. It explored how the crises changed management within the hospital and with external partners, how unintended consequences could be partly anticipated and mitigated, and how lessons learnt from the first impacted on managing the second. Based on our findings we propose a framework to identify and monitor unintended consequences inevitably occurring during crisis management. The topic of unintended consequences in healthcare should be further investigated in different settings, incorporating different perceptions and including not only decision makers but clinical frontline staff to optimize crisis management and minimize adverse consequences. Several future studies are needed to fill gaps in understanding on this topic. First, to assess what are the barriers and facilitators for improved learning processes between crises across various hospitals and departments. Secondly, since emergencies foster a collective focus on shared goals, how to maintain inter-institutional coordination between external stakeholders may contribute to improved understanding beyond the single center setting of this study. Finally, decision-makers and leaders should understand which factors may contribute to staff openness to change during crises, and transfer to routine periods. Understanding such complex phenomena will enable innovations, streamlined operations and improved care quality during both routine and crisis periods.

## Data Availability

The datasets presented in this article are not readily available because the data that has been used in the study is confidential. Requests to access the datasets should be directed to tomerbernstine@gmail.com.
