# Supplementary material for: Operating in recurrent crises: a qualitative study of decision-making and unintended consequences in a peripheral hospital
Source: Front Public Health. 2026 Mar 13;14:1728146. doi: 10.3389/fpubh.2026.1728146 (PMC13021871; doi:10.3389/fpubh.2026.1728146)
Supplement: Supplementary file 1 [file Table_1.docx]

**Supplementary**

**Table 1 Supplementary.** Theoretical constructs of *WHO’s Framework for Action* (6 Building Block) and its modifications

|  | *WHO’s Framework for Action* | Definition |
| --- | --- | --- |
| Building Blocks | Service Delivery | Good health services are those which deliver effective, safe, quality personal and non-personal health interventions to those who need them, when and where needed, with minimum waste of resources. |
|  | Leadership/ Governance | The governance overseeing and guiding the whole health system |
|  | Health workforce | Health workers are all people engaged in actions whose primary intent is to protect and improve health. |
|  | Medical products, vaccines & technology | Refers to the essential medical products, vaccines and technologies in a health system. |
|  | Information system | The generation and strategic use of information, intelligence and research on health and health systems, for decision-makers at different levels of the health system; |
|  | Financing | Adequate funds for health, in ways that ensure people can use needed services |
| Decision making process | Interaction between building blocks | Every action within the system influences the entire system, and the system influences every action. Related to the notion that any intervention targeting one building block will have certain effects (positive and negative) on other building blocks. |
|  | Decision making considerations | The design and eventual evaluation of any health system intervention must consider its possible effects across all major sub-systems of the health system. identifying and convening key stakeholders concerned with or affected by the intervention’s implementation is essential. They anticipate and hypothesize all possible ramifications of the intervention within each building block, while also thinking through the many interactions among the sub-systems. |
| Evaluation | Formative evaluation | An early-stage evaluation of the intervention (to fine-tune the intervention and adapt its implementation). To improve performance and to understand how the intervention really works given the characteristics of the system. |
|  | Summative evaluation | An advanced stage evaluation of the intervention. Interventions can be experienced differently; the impact evaluation should consider minimum and maximum effects. |
|  | Feedback | Systems are controlled by "feedback loops" that provide information flows on the state of the system, moderating behavior as elements react and "back-react" on each other. |
|  | Checkpoints | Adapt and redesign the proposed intervention to optimize synergies and other positive effects while avoiding or minimizing any potentially major negative effects |
| Context | Influence of outer impact | The connections and interventions in the health systems are affected not only by actions within the system but also outside the health system (such as national governance) and affecting healthcare decisions. |

**Table 2 Supplementary. Sample from Interview guide**

| **6 Building block construct** | **Sample question (lead)** |
| --- | --- |
| Description of the general role and areas of responsibility | Please describe your role in the hospital, what are your areas of responsibility:  During routine times  During the COVID pandemic era  During October 7th war period |
| Decision making process in routine and crisis | 1. Please describe the decision-making process of the organization's management during routine times before the pandemic (for example opening a new service in the hospital). 2. Please describe the decision-making process of the organization's management during the COVID pandemic. 3. Please describe the decision-making process of the organization's management during the October 7th war. |
| Checkpoints and feedback for intended and unintended consequences | 1. Between the opening and closing of the COVID department, did you conduct any evaluations on the functioning of the COVID department? 2. Did you conduct any evaluations of non-COVID departments (for example: Pediatric, Neurology, Cardiology) |
| Unintended consequences during COVID pandemic and war | Sometimes in the decision-making process, we aim for a specific outcome and additionally receive results that we do not expect; this phenomenon is also recognized in literature worldwide. Are you familiar with this phenomenon during routine/COVID/war? |
| Summative evaluation and consequences | What conclusions did you draw from the pandemic that are relevant to your area of responsibility? |
